# Nonlinear Fusion of Multispectral Citrus Fruit Image Data with Information Contents

**DOI:** 10.3390/s17010142

**Published:** 2017-01-13

**Authors:** Peilin Li, Sang-Heon Lee, Hung-Yao Hsu, Jae-Sam Park

**Affiliations:** 1 School of Engineering, University of South Australia, Mawson Lakes 5095, Australia; liypl001@mymail.unisa.edu.au (P.L.); Sang-Heon.Lee@unisa.edu.au (S.-H.L.); Hung-Yao.Hsu@unisa.edu.au (H.-Y.H.); 2 Department of Electronics Engineering, Incheon National University, 119 Academy Road, Yeon Su Gu, Incheon 22012, Korea

**Keywords:** image fusion, entropy filter, multiscale decomposition, wavelet transform, clustering

## Abstract

The main issue of vison-based automatic harvesting manipulators is the difficulty in the correct fruit identification in the images under natural lighting conditions. Mostly, the solution has been based on a linear combination of color components in the multispectral images. However, the results have not reached a satisfactory level. To overcome this issue, this paper proposes a robust nonlinear fusion method to augment the original color image with the synchronized near infrared image. The two images are fused with Daubechies wavelet transform (DWT) in a multiscale decomposition approach. With DWT, the background noises are reduced and the necessary image features are enhanced by fusing the color contrast of the color components and the homogeneity of the near infrared (NIR) component. The resulting fused color image is classified with a C-means algorithm for reconstruction. The performance of the proposed approach is evaluated with the statistical *F* measure in comparison to some existing methods using linear combinations of color components. The results show that the fusion of information in different spectral components has the advantage of enhancing the image quality, therefore improving the classification accuracy in citrus fruit identification in natural lighting conditions.

## 1. Introduction

For the robotic harvesting manipulator used in the horticultural industry, the main technique used to identify the location of fruits is a vision system. Since a vision system was proposed in this field [1], various sensor schemes have been practiced utilizing the intensity, the spectral information, or the laser range finder [2]. The successful fruit identification rate has been reported between 70% and 90% with a variation in laboratory conditions. Some issues are still to be solved before the widespread commercial use of the automatic harvesting manipulator. Normally, a color intensity thresholding with a certain filter has been used to contrast the salient features of an image [3]. In practice, the image data acquired by a single sensor is degraded since the imaging sensors have certain physical limitations. On the other hand, the light spectrum is potentially affected by multiple factors in an open unstructured environment [4]. In order to study the features from different wavebands on spectral coordinates, the hyperspectral techniques have been used to capture the necessary information from a wide range of the light spectrum. The study of the statistics of image segments obtained from different wavebands, in particular, techniques involving fuzzy wavebands, may provide the feature references necessary for the development of a machine vision system [5]. However, the distribution of the spectra on the segments of some components from certain spectral coordinates can be fuzzy and hence make the processing extremely difficult. Alternatively, multispectral image processing has been proposed to capture and combine more information with the capability of acquiring more wavebands utilizing a smarter processing method to improve detection [4,6]. Instead of dispersing the light spectrum into discrete wavebands, the multispectral image captures the specific range of wavelengths across wide spectral coordinates. For example, three charge coupled device (CCD) micro cameras have been used side by side with three different optical waveband filters of 550 nm, 650 nm, and 950 nm in the fruit harvesting [7]. The two ratios of the 550 nm to 950 nm filtered components and the 650 nm to 950 nm filtered components were calculated to contrast the fruits from the background. Successful detection was about 75% when the sky was overcast. In another study, a monochromatic near-infrared camera equipped with multi-waveband pass filters was used to identify unripe green citrus fruits from images [8] based on the measurements done on the green leaves and the green types of citrus fruits through seasons [4]. The reference index of band intensity was used followed by the global threshold to classify the citrus from the background. However, the resultant multispectral images were not well synchronized and aligned in dynamic scenes with the saturated area by the diffuse reflectance [4]. On top of that, the multispectral scheme has been broadly designed and practiced in research especially for analysis of inspection in agricultural applications. Various sensors are selected and practiced depending on the application particularity [9,10]. For example, image fusion improved fruit detection when the fruits in the visible image were over-exposed and the fruits in the thermal image were warmer than the canopy by using an infrared thermal camera with a digital color camera [11].

Multispectral image processing can be applied with multiple sensors shooting the same channel [9] or a single sensor shooting the channel by interchanging specific physical optical filters [10]. Basically, the linear combination of the components for different spectral regions are used to contrast the image for the subsequent segmentation application [12]. The coefficients in the linear combination can be found via the principal component (PC) analysis in the linear discrete analysis. The results show good classification accuracy using both near infrared spectroscopy and Fourier transform infrared spectroscopy in plant species [12]. In fact, the discriminant analysis using linear combination depends on the coefficients such as PCs which carries the optimal variance property. However, the result may not be accurate enough when the image data is distributed nonlinearly in color space, especially the image captured under the natural lighting condition. Hence, an alternative nonlinear solution using a fusion technique would be a way to find a more accurate discriminant result.

The multisensory fusion technique is a synergistic combination of different sources with complementarity to enhance the main information for various particular applications [13,14]. The use of image fusion technique has two issues to be addressed [13] namely the registration and the fusion of the data. The registration is the prerequisite of the fusion approach to align the different resource images precisely [13]. Most of the registration can be done by the algorithmic method to find a transform matrix based on the control point or the feature in the image [13] or using a customized frame for a region of interest [11]. However, the distortion in the image is transformed based on the availability of the information. The prevalent issue from the natural scene is the local disparity and the uncertainty of the availability of information which cannot be solved by the algorithmic global transform matrix, for example, the shifting of objects due to the difference in triggering a dynamic temporal coordinate [8]. In practice, when the registration of different resources is done, the further registration process can be possibly bypassed using portable multispectral imaging systems with a dual-band spectral or a three-band spectral imaging system [15,16]. A similar idea has been adopted in this research with more flexibility at the terminal of two sensors to acquire various citrus fruit image data online with some physical optical attenuation filter functions [17]. On top of the registration, the fusion technique itself is essential and applied in the signal level, pixel level, feature level, and symbol level approaches [13]. Signal level fusion performs the direct combination of several signals in order to provide a signal that has the same general format as the source signals. The pixel level fuses the images by determining the correlated pixel from each source image. Feature level fusion extracts the feature such as the edge or other feature and combines the feature into the fused image. Symbol level fusion processes the information at each source image and then makes a decision at a high level of abstraction to the fused image. In this research, the pixel level fusion is selected due to the fact that the main objective is to enhance the citrus fruit area based on the digitized spectra in intensity. The fusion of images aims to enhance the image quality while reducing more remaining background noise to find the area of the citrus fruits for the manipulator. The pixel-level fusion is a two-dimensional signal level fusion in two approaches namely the multiscale decomposition (MSD) and the non-multiscale decomposition (NMSD). The natural citrus fruit image data contains background noise with spatial variance properties such as the unstructured similar spectral reflection as citrus color on the side or tip of the leaves. Hence the MSD methods are suited to handle the fusion of two source images compared to the NMSD methods [13]. In consideration of both the spatial noise and the combination of different resources, the discrete wavelet transform (DWT) is adopted in this paper since the wavelet can deal with the frequency detail along the spatial coordinates in decomposition scale level. In DWT, the image is filtered by convolving two coefficient filters such as a low-pass and a high-pass filter both row wise and column wise successively. The original coefficient is downscaled to decompose the image into four coefficients such as low-low, low-high, high-low, and high-high pass coefficients in each decomposition scale level. The low pass approximation coefficient can be decomposed continually to the subsequent level. The issue of shift variance properties caused by the subsampling will not lead to extra distortion on the fused result if the images are well aligned by the nonparametric registration. In addition, the details with spatial property can be removed by thresholding the high pass coefficients in each scale level as in spatial coordinates. The fusion in the last level can be achieved with the combination of the envelope spectra from those low pass coefficients. The fusion of the coefficients from the multisensor has variant arithmetic rules such as maximum, minimum, mean or weighted mean, and majority voting in the multi-classifier application on high dimensional coordinate data space [18,19,20,21]. In addition the linear combination of component a* from CIE La*b* color space and component I from YIQ color space has been approached in a fusion strategy for robust tomato recognition [22]. Since in CIE La*b* color space, the red/green colors are represented along the a* axis, the extreme value along a* axis depends on the salient feature in the application. The extreme value is also limited by the constraints such as the citrus color and the feature from the background. However, when the image data is nonlinearly in the color space, the more robust method is encouraged instead of the linear combination.

The main purpose of this research is to investigate and evaluate a multispectral machine vision system which is robust to the background noise with spatial properties and the enhancement of the fundamental spectra from the images acquired from the multispectral sources. The alignment of two images in two coordinates is done with the customized hardware alignment. The synchronization is achieved by triggering two sensors in a master and slave architecture. Based on the hardware registration, the software-based nonlinear fusion rule has been proposed using information contents and color contrast in multiscale decomposition application using DWT. In DWT, the high pass coefficients are zeroed in order to remove the spatial unstructured noise. Since the removal of the detail in high pass coefficients blurs the area of the fruits in the resultant cluster image as well, the enhancement of the fundamental envelope spectra is considered in the fusion of two resources without losing the main information. Hence, the entropy from the color component with the color contrast and the homogeneity from NIR component is combined arithmetically to modify the original color image. As a result, the modified color image has better clustering results compared to the linear combination methods.

As follows, Section 2 summarizes the hardware registration followed by the detail of the nonlinear fusion approach with the information contents to modify the original color image. The modified color image is then classified with the selected clustering method for a nonlinear clustering problem. Section 3 evaluates the proposed fusion method using statistical harmonic *F* measure in comparison to the results of the other linear combination methods. The last section draws a conclusion for this paper.

## 2. Materials and Methods

### 2.1. Image Acquisition with a Registered Bi-Camera System

**Figure 1:**
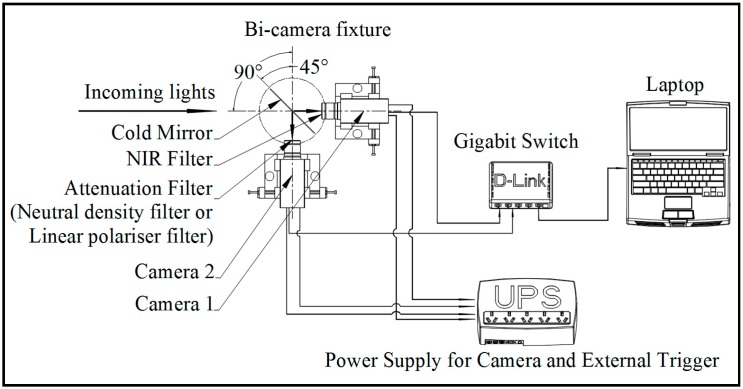
Assembly for citrus fruit image acquisition.

**Figure 2:**
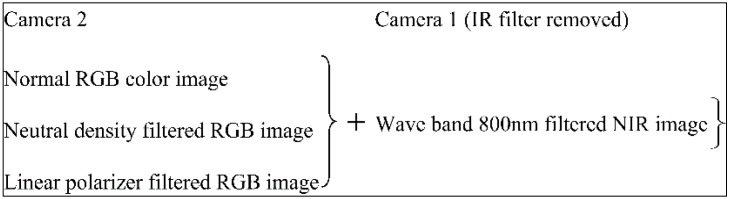
Combination of citrus fruit image acquisition.

A portable cold mirror and bi-camera assembly have been designed and prototyped for the acquisition of citrus fruit images under natural lighting conditions. Two cameras have been calibrated and synchronized in two coordinates namely the spatial coordinate and the temporal coordinate. The detail of calibration and alignment of two cameras refers to [17]. In order to align two input source images of RGB color model image (composed of three primary colors namely Red, Green, and Blue) and near infrared, two CCD cameras are aligned with a physical optical cold mirror in relative kinematic position in both spatial and temporal coordinates. The nonparametric registration is done by aligning two CCD cameras closely with two adaptors in spatial coordinates with 90° and each of both with 45° to the cold mirror. Then in the temporal coordinate, two cameras are synchronized with the embedded optocoupler interface to trigger two cameras via a master and slave architecture with the time difference faster than the minimum shutter speed of 10 μs. Due to the quality of the collinearity of the applied optical lens, the precision of alignment is achieved in pixel-level quantification. As a result, some pairwise pixels are well-aligned while the others have offset distributed sparsely in the superimposed image with average quantified offset about 10 µm. With this assembly, the setup for the image acquisition includes a personal computer, a gigabit Ethernet connector, and the UPS power supply for both cameras and two optocoupler interfaces as shown in Figure 1. The function of the cold mirror transmits the light with wavelengths over 700 nm and reflects the light with the wavelengths in the other visible areas. With a standard mounting interface, some physical optical filters are also interchanged at the terminal of two cameras, like near infrared (NIR) filter to replace the initial infrared filter on camera 1 and some attenuation filters on camera 2. The use of the second camera is to detect the gap of spectral reflectance function between the foreground fruits and the remaining background—namely, green leaves in a certain longwave area, for example, up to 900 nm [4]. Hence in the same pose, the different data can be captured, for example, the normal color image, the neutral density filtered image, and the linear polarizer filtered image, and each with their aligned NIR image as shown in Figure 2. The use of two filters such as the neutral density and the linear polarizer is to verify the image quality improvement. The applied neutral density filter reduces the light of all wavelengths to some extent without changing the color. The linear polarizer filter cuts off light wavelengths by polarization linearly. These filters can modify the density in a color space and reduce the detailed noise of the background.

The citrus fruit images are captured at the Alverstoke orchard on the Waite campus of the University of Adelaide under natural lighting conditions without any peripheral lighting sources. The time for photographing has been selected under different illumination conditions from sunny days to cloudy days. Every time the setup has been placed around the citrus canopy with a different direction to the sunlight including a shaded background. The only condition excepted is low illumination. The software HALCON is used and programmed to trigger and capture the pairwise images from two cameras synchronously. During the image acquisition, the setup is placed at a 1 to 2-m distance around the citrus canopy since the phase of “near to” is important to identify the citrus fruits for the automatic harvesting manipulator [23,24]. After the images are acquired, the preliminary study has been done to identify the issues to be addressed in the research [25]. From the preliminary study, the major issue is the nonlinearity of the image data in the color space. It is difficult to address this issue with some projection-based vegetation indices, linear discriminant analysis, or even hyperplane architecture or solutions with support vector machine. In addition, there is another quality issue where the remaining background contains some similar color to citrus fruits, especially on the side or tip of the leaves. This similar spectral noise normally has varied structures which make it difficult to remove with a certain morphological filtering with some proper size decision. From the preliminary research, it has been concluded that those background noises with spatial properties need to be resolved with a robust nonlinear multiscale decomposition method.

**Figure 3:**
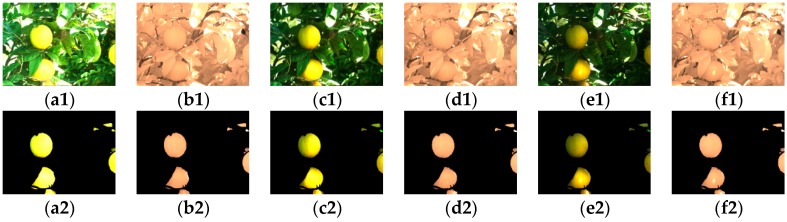
Segments of citrus fruit foreground for the calculation of standard deviation, (**a1**) is a normal color image (VIS); (**b1**) is the corresponding NIR image of (**a1**); (**c1**) is a neutral density attenuated image (NEUT); (**d1**) is the corresponding NIR image of (**c1**); (**e1**) is a linear polarizer-attenuated image (POLA); (**f1**) is the corresponding NIR image of (**e1**); (**a2**–**f2**) are the corresponding segmented images around the fruit area of the image from the first row respectively.

On the other hand, the NIR image has very little contrast between the fruit area and the remaining background. However, it is found that the NIR image can provide better homogeneity, especially around the fruit area due to the nature of lighting intensity. Figure 3 shows such differences. In the figure, the fruit area is segmented manually in RGB color image and the corresponding NIR image is also segmented using the same locations. For example, Figure 3(a1) is the normal color image without being modified by any filters (termed as VIS) and Figure 3(b1) is the corresponding aligned NIR image. The segmented images around the fruits areas are shown in the below in Figure 3(a2,b2) respectively. The color image modified with a neutral density attenuation filter (termed as NEUT) in Figure 3(c1) with its corresponding aligned NIR image in Figure 3(d1), and the color image modified with a linear polarizer attenuation filter (termed as POLA) in Figure 3(e1) with its corresponding aligned NIR image in Figure 3(f1) are presented in a similar way in Figure 3(c2–f2) respectively. To find the statistics such as standard deviation for the fruit area, on average 30 pairs of images for each type are selected randomly from the colored citrus fruit database since the data has covered various daytime illumination conditions.

**Figure 4:**
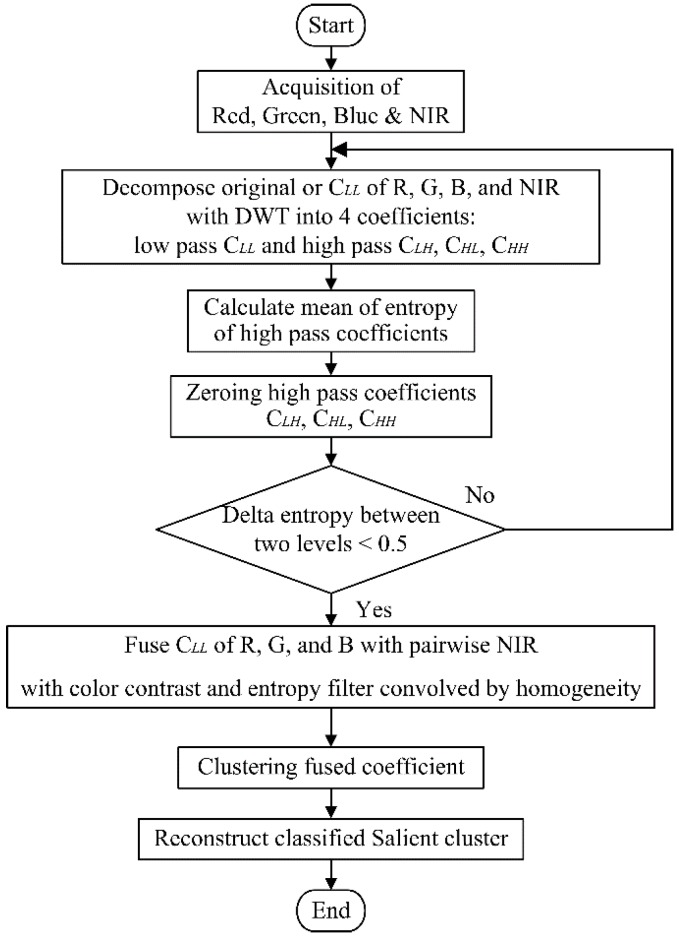
Scheme of fusion approach using RGB and NIR components.

The result in Table 1 and Table 2 show that the standard deviation of citrus fruits in the foreground area from NIR image are lower than from the original color image. The results indicate that, regardless of the featured background, the homogeneity of fruit foreground from NIR filtered image can be used in the fusion method to maintain the quality of a salient feature like citrus fruit area. If the part of NIR filtered image has higher contrast, the fusion of pairwise images can be enhanced further in the resultant fused image. As one of the main schemes of the proposed fusion method, the extra information with homogeneity from the NIR image is used to increase the contrast between the citrus fruit area and the remaining background area. The scheme of a nonlinear fusion approach is given graphically in Figure 4.

The nonlinear fusion is approached using discrete wavelet transform (DWT) on two different types of images as follows. Basically, the components of pairwise RGB and NIR images are decomposed into four coefficients including the low pass and the high pass coefficients. The high pass coefficients are zeroed to remove the noise in each DWT scale level. Since the high pass coefficients carry the detailed information, the entropy is measured on all high pass coefficients from RGB components. When the difference of this entropy between two continuous scale levels drops lower, two low-approximation coefficients are fused with entropy filter from RGB component convolved with the homogeneity from NIR component. Then the fused coefficient is clustered in low scaled resolution to find the salient feature cluster efficiently before the reconstruction of DWT.

### 2.2. Nonlinear Fusion of Two Images Using Discrete Wavelet Transform

This section describes how the high contrast property existed in the RGB color image and the homogeneity from NIR image are complementarily fused together using a discrete wavelet transformation. The nonlinear function is the main strength of the proposed scheme. When the homogeneity from the NIR image is fused into the RGB image, the high-frequency component—especially nonlinear noise—is significantly reduced for a more efficient clustering process. As mentioned before, the initial studies conducted in this project revealed that any linear combination of those two images would not provide reasonable results. The way the fusion is made is that the concept of entropy obtained from RGB images is convolved with the NIR images. By doing so, the uniformity of intensities around the target of citrus fruits are enhanced and, hence, the false positive existing around the parts of some leaves are possibly reduced from the true positive (citrus fruits) and clustered separately after the process. In the process, Daubechies wavelet of length 4 is used to decompose the images into the lower scale. Since in DWT, the high-frequency components contain unnecessary noise details, only the low-frequency components in each scale level are taken. With the theory of the orthogonal or biorthogonal mathematical properties [26,27] the construction of the discrete wavelet transform coefficient is based on the orthogonality condition to satisfy the Fourier transform of the basis coefficients [28]. The following is the DWT process with Daubechies wavelet of length 4 [29].

**Definition 1** (Daubechies four-term orthogonal filter)**.** *The low pass Daubechies wavelet four length filter is defined by the sequence $h_{f}=\left(h_{0},h_{1},h_{2},h_{3}\right)$, where h*_0_*, h*_1_*, h*_2_* and h*_3_ *are given by Equation (1) [28].*

(1) $$\begin{array}{l} h_{0}=\frac{1}{4\sqrt{2}}\left(1+\sqrt{3}\right)\;h_{1}=\frac{1}{4\sqrt{2}}\left(3+\sqrt{3}\right)\\ h_{2}=\frac{1}{4\sqrt{2}}\left(3-\sqrt{3}\right)\;h_{3}=\frac{1}{4\sqrt{2}}\left(1-\sqrt{3}\right) \end{array},$$

*The high pass filter is defined by sequence* $h_{\psi }=\left(g_{0},g_{1},g_{2},g_{3}\right)$ *and given by the rule* $g_{k}={\left(-1\right)}^{k}h_{3-k},\;k=0,1,2,3$. *So that the high pass coefficients have the form* $g_{0}=h_{3}$, $g_{1}=-h_{2}$, $g_{2}=h_{1}$, $g_{3}=-h_{0}$.

The filtered four coefficients, namely low-low (LL), low-high (LH), high-low (HL), and high-high (HH), can be expressed in the operation forms as expressed from Equation (2) to Equation (5), where “*” is the convolution operator [29].

(2) $$C_{LL}\left(j,m,n\right)=h_{\varphi }(-m)*h_{\varphi }(-n)*C\left(j-1,m,n\right),$$

(3) $$C_{LH}\left(j,m,n\right)=h_{\psi }(-m)*h_{\varphi }(-n)*C\left(j-1,m,n\right),$$

(4) $$C_{HL}\left(j,m,n\right)=h_{\varphi }(-m)*h_{\psi }(-n)*C\left(j-1,m,n\right),$$

(5) $$C_{HH}\left(j,m,n\right)=h_{\psi }(-m)*h_{\psi }(-n)*C\left(j-1,m,n\right),$$

**Figure 5:**
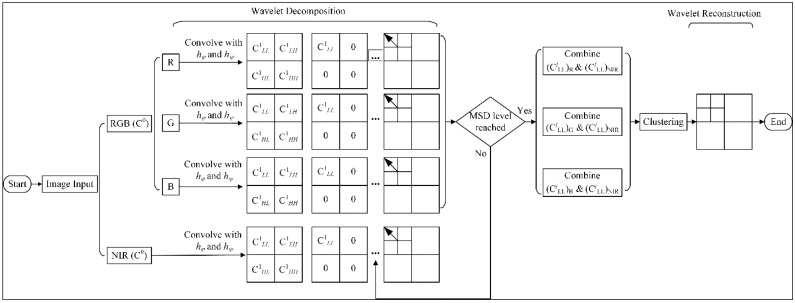
Fusion on color and near infrared images with DWT.

The process of DWT applied in the proposed fusion approach is given in Figure 5, where *h_ψ_*(.) and *h_φ_*(.) are high pass and low pass filter respectively in the wavelet transform matrix. In each level, three coefficients—namely C*_LH_*, C*_HL_*, and C*_HH_*—carry the high pass noisy details whilst the low pass coefficient C*_LL_* from NIR and RGB source images carry the envelope spectral information. Hence, zeroing those high pass coefficients can remove those unnecessary noisy details. The low pass coefficient carries the envelope information at the end. Then the fusion of the low coefficients from two source images can be calculated arithmetically to enhance the image quality. The enhancement around the salient feature is necessary and ideal for the subsequent clustering process.

**Figure 6:**
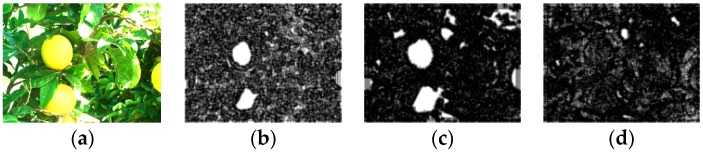
Entropy filter of RGB citrus fruit color image, (**a**) VIS color image; (**b**) Entropy filter of R component in level 3 of DWT; (**c**) Entropy filter of G component in level 3 of DWT; (**d**) Entropy filter of B component in level 3 of DWT.

The purpose of the fusion approach is to maintain or enhance the contrast around the features of interest. However, fusion rules such as mean average on both low pass filtered coefficients would blur the contrast of the entire image. From the preliminary study, it is found that the information represented by the entropy combined with the color intensity would be a good candidate for the fusion process. Figure 6 shows the entropy filter clearly. In Figure 6, the normal RGB (a) color image and (b–d) show the results obtained from the entropy filter with 3 by 3 window on the level 3 of DWT, respectively. The entropy filter applied in R and G components can provide very strong separation capability. Hence, the entropy filter is adopted in the proposed fusion rule.

Before the proposed fusion rule is introduced, the definition of entropy filter and the digital convolution operator are presented as follows.

**Definition** **2** [29]**.** *Using the concept of entropy, the entropy filter is defined as the average information within the neighborhood of each pixel in the image as follows.*

(6) $$E_{fitler}\left(X_{i,j}\right)=-\sum_{i-n,j-n}^{i+n,j+n}p\left(x_{i,j}\right)\log p\left(x_{i,j}\right),$$

*where X_i,j_ is the source image,* $p\left(x_{i,j}\right)$ *is the probability of the intensity level of the pixel in the image frame, 2n + 1 is the window size, and (i,j) is the coordinates of the current input pixel. The outside of the coordinate’s space is padded with zero during operation.*

**Definition** **3** [29]**.** *The convolution operator is defined as a linear spatial filtering in digital image processing. The convolution operator is defined for the pixel in the image* f(i,j) *and the mask from the second image* h(i,j)*, where (i j) is the coordinate of the pixel position.*

(7) $$h\left(i,j\right)*f\left(i,j\right)=\frac{1}{MN}\sum_{k_{1}=0}^{M-1}\sum_{k_{2}=0}^{N-1}f\left(k_{1},k_{2}\right)h\left(i-k_{1},j-k_{2}\right),\;\forall k_{1},k_{2}.$$

With these definitions, the following fusion rule is proposed to combine the NIR component and the RGB component to enhance the image quality.

**Definition** **4.** The fusion rule is an arithmetic addition of two separate parts as given in Equation (8). The first part is the low-low pass coefficient of RGB image obtained from DWT. This part represents the RGB color component with high contrast information maintained in the image. The second part is the convolution (represented by “*’) of two separate terms. $C_{LL/NIR}\left(i,j\right)$ *is the low-low pass coefficient of NIR image from DWT. This term contains high homogeneity, especially around the region of interest features (citrus fruits). The term* ${\left(h\left(i,j\right)\right)}_{E_{filter}(RGB)}$ *is the spatial mask of side 3 by 3 in the sliding window in the resultant image obtained after the entropy filter applied to C_LL_ of RGB coefficient and* J(i,j) *is a corresponding mask of all 1s with the same size of* ${\left(h\left(i,j\right)\right)}_{E_{filter}(RGB)}$*. Here, (i,j) represents the coordinate of a pixel in the image obtained after a DWT is applied. Note that, by combining these two terms with the convolution operation, the low contrast between citrus fruits and the background are completed with the entropy filtered image. The resulting coefficient has enhancement of contrast with high homogeneity in the clustered region.*

(8) $$C^{\prime }\left(i,j\right)=C_{LL/RGB}\left(i,j\right)+\left[J\left(i,j\right)-{\left(h\left(i,j\right)\right)}_{E_{filter}(RGB)}\right]*C_{LL/NIR}\left(i,j\right).$$

**Figure 7:**
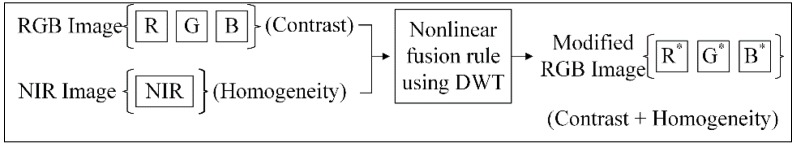
Modification of the original RGB color image with the corresponding NIR image using the proposed fusion approach.

With the addition of the convolution result to the low-low pass coefficient of RGB image, the region around the citrus fruits in the image would have enhanced contrast thanks to the entropy filter with higher homogeneity from the NIR coefficient. Fundamentally, the proposed fusion rule takes advantages of high contrast provided by RGB color component and high homogeneity provided by NIR image with a use of entropy filter. If the NIR has high contrast, the convolution part will contribute to the enhancement of the modified color image with both the homogeneity and the contrast from the longwave area. As shown in Figure 7, the nonlinear fusion approach combines the homogeneity from NIR component and the contrast of color component into a modified color image with quality enhanced.

**Figure 8:**
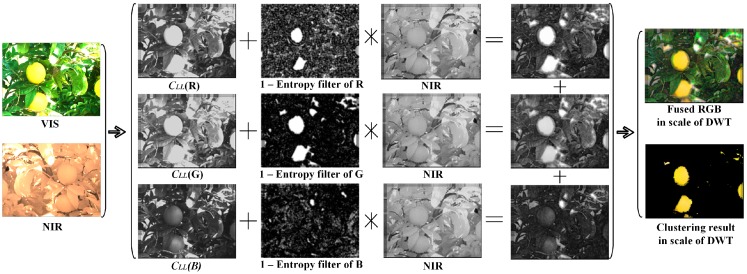
Fusion of two aligned images using RGB components with fusion rule.

In addition, an example is given graphically in Figure 8 to illustrate the whole procedure succinctly. As shown, two aligned images RGB and NIR are decomposed with DWT to a certain scale level with all high pass coefficients zeroed. The entropy of each R, G, and B from the color component are convolved with the NIR then added back to the components of R, G, and B respectively to create a modified color component to form a new enhanced color image for the subsequent clustering process.

### 2.3. Clustering Fused Image with C-Means Algorithm

The fused image is normally identified as a multiclass nonlinear classification problem based on the preliminary study. The fused color coefficient is then processed with nonlinear clustering method to separate the citrus fruit area from the remaining background using statistical C-means clustering algorithm. The C-means clustering algorithm is adopted as the competitive learning based partitioning method using the hyperspherical metrics [30]. The centroids of the hyperspheres represent the label of each cluster with a clear color contrast to the neighboring regions. The detail of the algorithm refers to [31]. On the use of C-means clustering algorithm, the initial value for the number of clusters is estimated by trial and error to provide the best result for each image in this work. Once the centroids of clusters are converged, the value of refined centroids is used to classify the citrus fruits from the remaining background. Finally, the resultant cluster is used as the final coefficient image to be reconstructed back to the original resolution as shown in Figure 8. Then the resultant image is evaluated by comparing to the segmentation reference using a statistical measure.

## 3. Evaluation and Results Discussion

The validation for the classified result is non-trivial since there is no benchmark for the dissimilarity comparison. Also, the structure of the image data in color space is unknown a priori. In addition, the outcome of clusters from one image is completely independent of the others. Hence without universal reference, the citrus fruit area is manually segmented in advance. Then the classified cluster with citrus fruits is utilized in the evaluation study since the remaining background is of no interest.

To measure the accuracy of the clustering, *F* measure is adopted as a harmonic mean using the fraction of the precision and the recall [32]: the precision (*P*) is the fraction of retrieved objects that are relevant while the recall (*R*) is the fraction of relevant objects that are retrieved.

(9) $$P=\frac{TP}{TP+FP},\;R=\frac{TP}{TP+FN},$$

where a true positive (*TP*) assigns two similar objects to the same cluster. There are two types of errors: a false positive (*FP*) assigns two dissimilar documents to the same cluster and a false negative (*FN*) assigns two similar documents to different clusters. In this case, since the background clusters are unknown with unstructured fluent features, only the salient cluster with citrus fruits is selected to compare with the weak manual segmentation reference. In this way, each pixel is valued first. In each cluster, the pixels belong to the other clusters are labeled by zero. Hence, the salient non-zero pixels are used to calculate the components for *F* measure. By following the definition, FP detects the background pixels which are not included in the reference. FN rejects the foreground pixels, which are included in the reference, into the background clusters. Assume the reference image by *R* and the clustering result by *C* with non-zeros pixels for citrus fruits, the true positive TP is found by the binomial coefficient of R∩C, FP is found by the binomial coefficient of C−R∩C, and FN is found by the binomial coefficient of R−R∩C. Then, the *F* measure is calculated with weight parameter. Note that *P* is the measure of the amount of false positive and if it is 1, then there is no false positive, i.e., no background pixels are included in the foreground. On the other hand, *R* is a similar measure for false negative and if it is 1, then there is no false negative, i.e., no foreground pixels are included in the background. The *F* measure is the weighted harmonic mean of precision and recall:

Then the *F* measure is the weighted harmonic mean of precision and recall.

(10) $$F=\frac{1}{\alpha \frac{1}{P}+(1-\alpha )\frac{1}{R}}=\frac{(\beta^{2}+1)PR}{\beta^{2}P+R},$$

where $\beta^{2}=\left(1-\alpha \right)/\alpha$ and α∈[0,1] or thus $\beta^{2}\in \left[0,\infty \right]$. The balanced *F* measure weighs the precision and the recall equally with α=0.5 or β=1. In this application, the precision is more important than the recall—i.e., the false positive in *P* will decide the accuracy of clustering. Ideally, the result should have the least amount of background noise classified as the salient feature (i.e., the reduction of false positive). The measure of a false negative is not as important as the value of a false positive since simply the main purpose of the clustering is the detection of the majority of citrus fruit area and a small number of pixels excluded from the salient feature cluster will not create issues in the practical use of the proposed algorithm.

With these performance evaluation metrics, the proposed nonlinear fusion method is evaluated using a comparison study with a number of existing methods. Since the proposed method is developed for the agricultural application, naturally the first method employed is the projection based vegetation color indices [33], i.e., the projection $w^{T}x$ from the coordinates of the RGB color space in **w**, where $w^{T}=\left(R,G,B\right)$ onto the lower dimensional space of **x** where **x** is a direction vector. The direction vector is constructed by the coefficients of the color indices or the normalized indices such as the applied two color indices R-B and 2R-G-B. Since the vegetation color index can be interpreted as a linear combination of color components, another linear discriminant analysis method, Fisher linear discriminant analysis (FLDA) [34,35], as a supervised linear discrimination analysis is also adopted in the study. FLDA is the dimension reduction technique. Two segments, namely citrus fruit foreground and the remaining background, are manually segmented to find the maximal eigenvector. The projection data is classified using the nearest neighbor based on the centroids of two prior segments. On top of FLDA, principle component analysis (PCA) is adopted to find a direction which provides the maximum variance between the data sets in two different ways, one with R, G, and B components only and the other with R, G, B, and NIR components as well. PCA automatically extracts the eigenvectors based on the maximization of the variance of the projected data using the centered covariance matrix of the data [36].

The evaluation study is conducted with MATLAB (8.6.0.267246 (R2015b)) on a computer of the 64-bit operating system with Intel(R) Core(TM) i7-4790 CPU @ 2.40 GHz and 4 GB RAM. Two vegetation color indices, “R - B” and “2R - G - B” and two approaches with PCA and FLDA are applied in addition to the proposed method in the study. In the color indices algorithm, the projected data is classified into two segments using the optimal thresholding. In PCA, two ways of linear combinations of intensities are used; one with only R, G, and B color components and the other with NIR in addition to those three color components to have a fair comparison with the proposed method. The first eigenvector is determined based on the centered covariance matrix using the components to be combined. The projected data are classified using the optimal global thresholding. In addition, FLDA with a manual segmentation is applied in the study. For comparison of different methods, the clusters with citrus fruits are separated and the *F* measure is calculated to check the dissimilarity. In the dissimilarity measure, the ground truth references are manually created a priori. Only the objects of interest, i.e., areas around citrus fruits, are segmented manually and used for the *F* measure calculation. The methods mentioned above are applied to the original images obtained directly from the cameras and also to the images after the DWT has been applied, except that FLDA is applied only to the original color image.

**Figure 9:**
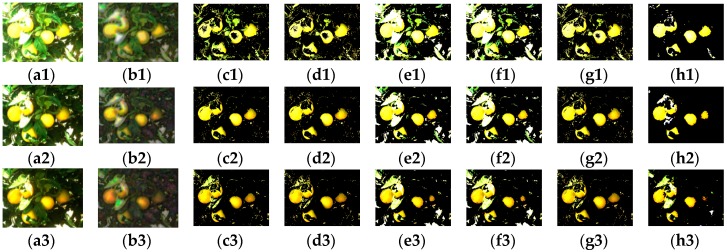
Example of validation, (**a1**) is normal color image (VIS); (**b1**) is scaled modified color image of (**a1**); (**a2**) is neutral density attenuated color image (NEUT); (**b2**) is modified color image of (**a2**); (**a3**) is linear polarizer attenuated color image (POLA); (**b3**) is modified color image of (**a3**); (**c1**–**c3**) are the results by ‘R-B’ for images (**a1**–**a3**); Respectively (**d1**–**d3**) are the results by ‘2R-G-B’; (**e1**–**e3**) are the results by PCA using R, G, and B; (**f1**–**f3**) are the results by PCA using R, G, B, and NIR component; (**g1**–**g3**) are the results by FLDA; (**h1**–**h3**) are the results by the nonlinear fusion method.

**Figure 10:**
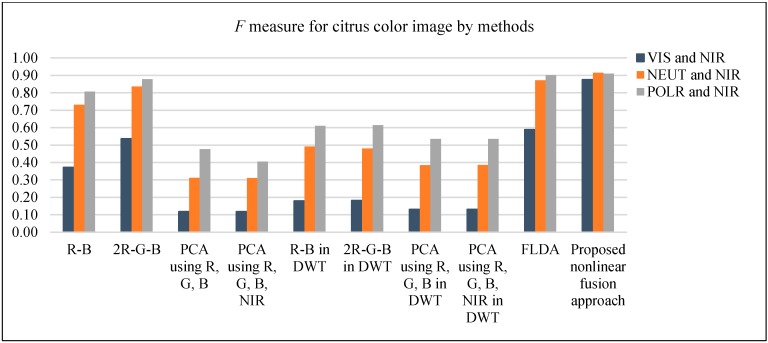
*F* measure for citrus fruit cluster image by different methods.

Figure 9 shows an example of the results of the study. Figure 9(a1–a3) display the three types of color images, Figure 9(b1–b3) are the corresponding modified color images by nonlinear fusion approach after DWT applied, The images listed in columns from 3rd column to 7th column in Figure 9 are the results obtained by the methods of “R-B” color index, “2R-G-B” color index, a linear combination of R, G, and B only; a linear combination of R, G, B, and NIR; and the FLDA, respectively. Figure 9(h1–h3) shows the results of the proposed algorithm. In the figure, the color image in the first row is the image with no filters (termed as VIS) and the second row is the one with a neutral density filter (termed as NEUT) and the one in the third row is with linear polarizer filter (termed as POLA), respectively. These two additional filtered color images are employed to the study to show the effects of the proposed algorithm. The figures clearly show the proposed algorithm presents the robust results with high reduction of background noise, especially with the images of no filters. This can be observed more clearly in Table 3 and Figure 10, which shows the results of the average *F* measures obtained from 30 randomly selected images each with no filter, with a neutral density filter, and with a linear polarizer filter. The corresponding pairwise images of NIR are also used together with those images. The *F* measures of the VIS images show the superior performance of the proposed scheme compared to other methods, while for NEUT and POLA images, the proposed method shows a bit better performance than some of the existing methods. The reason is that these two types of images have been attenuated with the filter which makes the nonlinear noises reduced compared to the VIS images. However, Table 3 shows the performance as the quantified measure, but in reality, in the image, the proposed method can provide a significant reduction of the false positive background noise, which is more desirable. The proposed fusion rule enhances the salient feature to complement the loss of content contrast by thresholding the high pass coefficients in DWT. In addition, the identification rate of applied methods has been counted. Since the focus is the colored citrus clustering, and due to the varied illumination condition and the non-Euclidean structure of the citrus fruit, only the number of visible colored citrus fruits are counted by comparison to the resultant cluster. The quantified rate by the vegetation indices are similar and so is the linear combination of color components with or without the NIR component. The rate by the proposed method is acceptable as shown in Table 4 but with high *F* measure for each type of color image by comparing to the other methods and to the results by FLDA comparably, especially with low false positive in *F* measure for the normal color image.

Without loss of the salient feature information, the proposed nonlinear fusion method is more robust against the false positive fraction. As expected, since the computational complexity of the proposed method is quite high due to the processes involved in the decomposition, clustering of the fused image data, and the reconstruction of DWT, the processing time would be substantially increased compared to other methods (See Table 5). However, the efficiency is compromised with the accuracy of the application at the end. The overall performance shows that the proposed nonlinear fusion method has advantages in the development of a machine vision system for an automatic fruit harvesting manipulator.

## 4. Conclusions

The information contents have been approached with a fusion technique in the multispectral citrus fruit image data classification. The nonlinear fusion of salient features from the low pass decomposed coefficients with the high pass coefficients thresholded in DWT improves citrus fruit detection under the natural illumination condition. The combination of the homogeneity from the near infrared source image and the complementary entropy filter from the color source image in the arithmetic fusion rule has both color contrast reservation and enhancement. The modification of a color image with the fusion of information contents has been evaluated with statistical harmonic *F* measure in comparison to the vegetation indices and the linear combination of the components from the color image and the near infrared image. The results show that the proposed nonlinear fusion method is more robust compared to other methods considered. Therefore, the fusion of multispectral source images would be a promising alternative in the development of a machine vision system for the automatic harvesting manipulator.

## Figures and Tables

**Table 1:** STD of RGB components for types of color images.

Types of Color Image	VIS			NEUT			POLA		
Components of RGB color space	Red	Green	Blue	Red	Green	Blue	Red	Green	Blue
STD of citrus foreground in color	0.1454	0.1625	0.2037	0.2440	0.2494	0.1338	0.2632	0.2397	0.1034

**Table 2:** STD of intensity of NIR component pairwise for types of color images.

Intensity of NIR for Color Image	Intensity of NIR with VIS	Intensity of NIR with NEUT	Intensity of NIR with POLA
STD of citrus foreground in NIR	0.1336	0.1275	0.1306

**Table 3:** *F* measure for classified results with different linear methods and nonlinear fusion method.

*F* Measure by Methods (*α* = 0.98–0.99)			
Methods	VIS	NEUT	POLA
R-B	0.3723	0.7318	0.8062
2R-G-B	0.5363	0.8365	0.8785
PCA using R, G, and B	0.1179	0.3115	0.4765
PCA using R, G, B, and NIR	0.1179	0.3107	0.4049
R-B in DWT	0.1793	0.4918	0.6104
2R-G-B in DWT	0.1824	0.4797	0.6143
PCA using R, G, and B in DWT	0.1303	0.3853	0.5349
PCA using R, G, B, and NIR in DWT	0.1303	0.3854	0.5350
FLDA	0.5881	0.8715	0.9009
Proposed nonlinear fusion method	0.8753	0.9147	0.9099

**Table 4:** Identification of colored citrus in resultant cluster.

Methods	(R-B)/(2R-G-B)	PCA Using (R, G, B)/(R, G, B, NIR)	(R-B)/(2R-G-B) in DWT	PCA Using (R, G, B)/(R, G, B, NIR) in DWT	FLDA	Proposed Nonlinear Fusion Approach
Identification (%)	0.877	0.864	0.890	0.882	0.917	0.882

**Table 5:** Processing time (second) for different classification methods.

Methods	R-B	2R-G-B	PCA Using R, G, B	PCA Using R, G, B, NIR	FLDA	Proposed Nonlinear Fusion Approach
Processing time	0.29	0.28	0.63	0.67	1.27	10.13
